# Generation and Characterisation of a Canine EGFP-HMGA2 Prostate Cancer *In Vitro* Model

**DOI:** 10.1371/journal.pone.0098788

**Published:** 2014-06-10

**Authors:** Saskia Willenbrock, Siegfried Wagner, Nicola Reimann-Berg, Mohammed Moulay, Marion Hewicker-Trautwein, Ingo Nolte, Hugo Murua Escobar

**Affiliations:** 1 Small Animal Clinic, University of Veterinary Medicine Hannover, Hannover, Germany; 2 Institute of Biophysics, Leibniz University Hannover, Hannover, Germany; 3 Department of Pathology, University of Veterinary Medicine Hannover, Hannover, Germany; 4 Division of Medicine, Haematology, Oncology and Palliative Medicine, University of Rostock, Rostock, Germany; Innsbruck Medical University, Austria

## Abstract

The architectural transcription factor HMGA2 is abundantly expressed during embryonic development. In several malignant neoplasias including prostate cancer, high re-expression of *HMGA2* is correlated with malignancy and poor prognosis. The *let-7* miRNA family is described to regulate *HMGA2* negatively. The balance of *let-7* and *HMGA2* is discussed to play a major role in tumour aetiology. To further analyse the role of HMGA2 in prostate cancer a stable and highly reproducible *in vitro* model system is precondition. Herein we established a canine CT1258-EGFP-HMGA2 prostate cancer cell line stably overexpressing HMGA2 linked to EGFP and in addition the reference cell line CT1258-EGFP expressing solely EGFP to exclude EGFP-induced effects. Both recombinant cell lines were characterised by fluorescence microscopy, flow cytometry and immunocytochemistry. The proliferative effect of ectopically overexpressed HMGA2 was determined via BrdU assays. Comparative karyotyping of the derived and the initial CT1258 cell lines was performed to analyse chromosome consistency. The impact of the ectopic *HMGA2* expression on its regulator *let-7a* was analysed by quantitative real-time PCR. Fluorescence microscopy and immunocytochemistry detected successful expression of the EGFP-HMGA2 fusion protein exclusively accumulating in the nucleus. Gene expression analyses confirmed *HMGA2* overexpression in CT1258-EGFP-HMGA2 in comparison to CT1258-EGFP and native cells. Significantly higher *let-7a* expression levels were found in CT1258-EGFP-HMGA2 and CT1258-EGFP. The BrdU assays detected an increased proliferation of CT1258-HMGA2-EGFP cells compared to CT1258-EGFP and native CT1258. The cytogenetic analyses of CT1258-EGFP and CT1258-EGFP-HMGA2 resulted in a comparable hyperdiploid karyotype as described for native CT1258 cells. To further investigate the impact of recombinant overexpressed HMGA2 on CT1258 cells, other selected targets described to underlie HMGA2 regulation were screened in addition. The new fluorescent CT1258-EGFP-HMGA2 cell line is a stable tool enabling *in vitro* and *in vivo* analyses of the HMGA2-mediated effects on cells and the development and pathogenesis of prostate cancer.

## Introduction

According to recent global cancer statistics, prostate cancer is the second most frequent diagnosed cancer and sixth leading cause of death among males in economically developed countries [1]. Besides man, the dog is the only known domesticated mammalian species developing spontaneous prostate cancer with considerable interest [2].

Unlike the situation in men, the incidence of canine prostate carcinomas is low accounting for 0.2 to 0.6% of canine neoplasias [3]. However, the disease is locally invasive in both species with a comparable progression, metastatic pattern and histopathology [2], [4].

The mean age at diagnosis in dogs is ten years and thus, predominantly affecting elder individuals as it is also reported in men [5]–[7]. Considering the physiologic age at prostate cancer diagnosis, the respective life span is similar between the two species showing increased incidence with age [6].

In humans, prostate cancer is usually a rather slow-progressing cancer whereas canine prostate cancer is growing rapidly, highly aggressive and less differentiated presenting a poor prognosis [3], [8]. Cancer of the canine prostate gland is unresponsive to androgen withdrawal therapy resembling mostly human poorly differentiated, androgen refractory prostate cancer [4], [9]. Due to the similarities concerning the presentation of human and canine prostate cancer, the dog has lately been focused as useful natural complementary animal model for evaluating novel prostate cancer therapies [10].

Early detection of prostate cancer in men is currently being done using established biochemical molecular markers such as prostate specific antigen (PSA) and prostate specific membrane antigen (PSMA) with considerable success.

In comparison to the situation in humans, in dogs prostate cancer is diagnosed at a very late disease stage due to the absence of reliable prostate-specific biochemical prognostic marker tools and the treatment remains palliative since still no standard therapeutic approach for treatment of canine prostate cancer is available [11], [12]. Although several studies report immunoreactivity for human PSA in canine non-neoplastic prostate tissue and prostate cancer, up to now PSA could not be found in the plasma of prostate cancer bearing dogs [9], [12]–[16].

Consequently, the identification of reliable molecular biomarkers, such as PSA and PSMA in men, allowing an early detection and reliable prognosis of canine prostatic cancer would be of significant value for future development and evaluation of therapeutic strategies as well as the assessment of treatment response [2].

In this context the High-Mobility-Group Protein A2 (HMGA2) was recently found to serve potentially as a prognostic marker for canine prostatic neoplasias [17]. Herein, the analysis of a subset of different canine prostate tissue samples clearly showed that expression of *HMGA2* increases significantly in correlation to the malign grade of the tissue samples [17]. Furthermore, *HMGA2* was found to serve as a potential differentiation marker of canine malignant T- and B-cell lymphoma [18] and to be strongly upregulated in canine oral squamous cell carcinoma (unpublished data).

In humans, a re-expression of *HMGA2* was also found in various malignant tumours such as leukaemia [19], [20], lymphoma [18], mammary [21], pancreas [22], non-small cell lung [23], oral squamous cell [24], and thyroid carcinoma [25] being an indicator of poor prognosis. In a recent study, the HMGA2 protein expression was demonstrated to be significantly higher in tumour tissues compared with adjacent normal tissues [26]. In addition, an *HMGA2* involvement in the induction of epithelial-to-mesenchymal transition (EMT) in the human prostate cancer cell line PC-3 was found [26].

These findings suggest that HMGA2 plays a central role in different tumour entities including prostate cancer within both species strongly supporting *HMGA2* re-expression as a prognostic tumour marker.

In general, the highly conserved HMGA2 protein is abundantly expressed during embryonic development acting as an architectural transcription factor in the nucleus [27], [28]. Within this role, HMGA2 is widely reported to be involved in a variety of cellular processes such as gene expression, induction of neoplastic transformation, and promotion of metastasis [29], [30].

The expression of *HMGA2* is regulated via micro RNAs (miRNA) of the *let-7* family by binding to sequences located in the 3′ untranslated region (UTR) of the transcript [31]–[35], all of which are conserved in rodents, dog, and chicken [36]–[38]. Binding of *let-7* miRNAs to complementary sequences regulates post-transcriptionally the expression of *HMGA*2 in a negative way [31], [35], [39], [40]. Recently a deregulated *let-7* expression was associated with lung [41], [42], breast [43] and prostate cancer [44].

The canine prostate adenocarcinoma derived cell line CT1258 [45]–[47] used within the present study was also analysed for *HMGA2* marker expression by us revealing a strong overexpression (unpublished data). This result allows to hypothesise that an overexpression of this target gene is likely to play an important role in canine prostate cancer, promoting the proliferation of tumour cells.

To verify this hypothesis, the availability of stable tools allowing evaluating the described *HMGA2*-*let-7* axis in prostate cancer *in vitro* and *in vivo* is precondition. Therefore we established stably transfected cell lines of CT1258 providing a reliable *in vitro* system to analyse the key aspects of our hypothesis.

We analysed the proliferative effects of abundantly expressed recombinant *HMGA2* on CT1258 cells. Therefore, a stable CT1258 cell line expressing recombinant EGFP-tagged HMGA2 (CT1258-EGFP-HMGA2) was generated using an expression vector construct containing the coding sequence (CDS) of the canine *HMGA2* gene lacking the 5′UTR and 3′UTR and therefore not underlying the direct negative regulation mechanisms by *let-7* [31].

To assess the functionality of the recombinant HMGA2 expression vector and to monitor the biological activity of the recombinant expressed HMGA2, a GFP-tag was added to the *HMGA2* CDS generating a HMGA2-GFP fusion protein. To exclude that the GFP protein has an effect on cell proliferation, a further stable CT1258 cell line (CT1258-EGFP) expressing solely GFP was generated. The *HMGA2* and *let-7a* expression was determined via quantitative real-time PCR in CT1258-EGFP-HMGA2 and CT1258-EGFP in comparison to native CT1258 cells.

Additionally, the expression of selected direct and indirect HMGA2-targets such as *HMGA1* [48], *SNAI1* [49], *SNAI2* and *CDH1* [49] was analysed.

To characterise the proliferation of the described three cell lines, BrdU incorporation assays were performed. Comparative karyotype analyses of the newly generated and the initial CT1258 cell lines were additionally carried out to identify cytogenetic changes possibly occurring during plasmid integration into the genome of CT1258 during the establishment of the stable recombinant cell lines.

In summary the new fluorescent canine CT1258-EGFP-HMGA2 cell line provides a valuable tool for further investigations on HMGA2-mediated proliferative effects and HMGA2 regulation mechanisms elucidating the development and pathogenesis of canine prostate cancer. As the dog represents a unique natural model for human prostate cancer, the insights concerning the involvement of HMGA2 in canine prostate cancer will provide benefit for both, humans and dogs, concerning the development of therapeutic strategies and the assessment of the treatment success.

## Methods

### CT1258 Cell Line

The cell culture conditions, as well as the characteristics of the canine prostate carcinoma cell line CT1258 have been described previously by us [45], [46].

### pEGFP-C1-*HMGA2* Expression Plasmid

The protein coding sequence of the canine *HMGA2* was amplified by PCR using primer pair EcoRI_sA2_lo (5′-CGGAATTCCTAGTCCTCTTCGGCAGACT-3′), BamHI_sA2_Up (5′-CGGGATCCCACCATGAGCGCACGCGGT-3′). The obtained PCR products were separated on a 1.5% agarose gel, recovered with QIAquick Gel Extraction Kit (QIAGEN, Hilden, Germany), ligated in the pEGFP-C1 vector plasmid (BD Bioscience Clontech, Palo Alto, CA, USA) and sequenced for verification. Transfection with the pEGFP-C1-*HMGA2* construct leads to the expression of a recombinant EGFP-HMGA2 fusion protein which is expected to be localised in the nucleus.

### Generation of Fluorescent CT1258 Cell Lines

#### Transfection of CT1258 cells

300,000 native CT1258 cells were seeded in 6-well plates 24 hours prior transfection and cultivated at standard conditions using medium 199 (Life Technologies GmbH, Darmstadt, Germany) supplemented with 10% FCS (PAA Laboratories GmbH, Coelbe, Germany), and 2 % penicillin/streptomycin (Biochrom AG, Berlin, Germany).

The transfection was performed according to the manufacturer’s instructions using 7.5 µl Mirus TransIT-2020 reagent (Mirusbio LLC, Madison, WI, USA) in 250 µl serum-reduced Opti-MEM I medium (Life Technologies, Darmstadt, Germany) containing 2.5 µg of pEGFP-C1 (BD Bioscience Clontech, Palo Alto, CA, USA) or recombinant pEGFPC1-HMGA2 plasmid. After treatment, the cells were incubated for 24 hours in the culture media. The uptake and expression of DNA was verified by fluorescence microscopy using a Leica DMI 6000B fluorescence microscope (Leica Microsystems GmbH, Wetzlar Germany).

#### G418 selective antibiotic kill curve assay

Prior generation of the fluorescent CT1258 cell lines, the titration of the proper amount of the selective antibiotic G418 (syn. Geneticin; Life Technologies, Darmstadt, Germany) required for selection of CT1258 cells was carried out with a kill curve assay. Different G418 concentrations were applied (0, 100, 200, 400, 600, 800, 1000 µg/ml) on 100,000 native CT1258 cells seeded in the wells of a 12-well plate. For selection of positive cells after transfection the concentration was used in which no cell survived the upper conditions after seven days.

#### Selection of positively transfected CT1258 cells

Fluorescent variants of the cell line CT1258 were established to constitutively express the enhanced green fluorescent protein (EGFP) encoded by the empty pEGFP-C1 plasmid and an EGFP-HMGA2 fusion protein by expression of the recombinant pEGFP-C1-*HMGA2* plasmid. To establish the stable CT1258 cell lines, the transfected cells were selected with the antibiotic G418 (Life Technologies, Darmstadt, Germany).

Initially a concentration of 400 µg/ml G418 in the medium was used when selecting for stable cells. One day after transfection, the cultivation medium 199 was replaced with medium 199 containing G418. Subsequently the selection medium was changed each 24 to 48 hours for the first two weeks which leads to the selection of cells that have stably incorporated the GFP plasmid with the encoded antibiotic resistance gene neomycin for selection in mammalian cells into their genomic DNA. Cells not expressing the construct will be killed by G418. The concentration of G418 was lowered to 300 µg/ml after three months of consistent selection for maintenance of the generated fluorescent cell lines.

### Fluorescence Microscopy and Flow Cytometry (FCM)

GFP expression of the fluorescent cell lines CT1258-EGFP and CT258-EGFP-HMGA2 was analysed after G418-selection by fluorescence microscopy and quantified in a FACSCalibur flow cytometer (Becton, Dickinson and Company, Heidelberg, Germany) with the FL-1 channel to determine the percentage of GFP-positive cells. Cells were trypsinised for 3–5 min, resuspended in BD FACSFlow Sheath Fluid (Becton, Dickinson and Company, Franklin Lakes, NJ, USA) containing 1 µM TO-PRO-3 (Life Technologies GmbH, Darmstadt, Germany), and at a total number of 1×10^4^ events was measured for each sample by flow cytometry. TO-PRO-3 is a far-red cell impermeant nucleic acid stain measured in the FL-4 channel allowing ultrasensitive detection of double-stranded DNA of dead cells. The analysis of the flow cytometry data was done using with Cell Quest software (Becton, Dickinson and Company, Franklin Lakes, NJ, USA).

### Immunocytochemistry

#### Embedding of the cell lines

Cell suspensions of cultured cell lines were fixed in 4% formalin. Cell pellets obtained by centrifugation were embedded in paraffin and cut in 3–4 µm slices for immunocytochemical staining.

#### Immunocytochemical staining

For antigen retrieval, microwave heating of paraffin sections in 0.01 M citric acid buffer (pH 6.0 for 20 min) (Quartett, Berlin, Germany) was performed. The inhibition of endogenous peroxidase activity was achieved by immersion in 0.5% H_2_O_2_ (v/v) in methanol (20 min). After draining the blocking serum the sections were incubated with a polyclonal goat anti-human HMGA2 antibody (R & D Systems, Minneapolis, MN, USA) diluted 1∶400 in phosphate-buffered saline (PBS, pH 7.2, 0.15 M) approximately 16–18 h at 4°C. After washing in PBS, the sections were incubated with a biotin-conjugated antibody to goat IgG (Vector Laboratories, Burlingame, CA, USA). The avidin-biotin-peroxidase reagent (Vector Laboratories) was applied according to the manufactureŕs instructions. The chromogen used was 3′3-diaminobenzidine-tetrahydrochloride (Sigma Aldrich, München, Germany) 0.05% (w/v) with 0.03% H_2_O_2_ (v/v) as substrate in 0.1 M Tris-buffered saline (Tris-hydroxymethyl-aminomethane; Merck, Darmstadt, Germany). The sections were counterstained with Mayeŕs haematoxylin and mounted. Negative controls were performed by replacing the primary antibodies by normal goat serum. For establishing the immunocytochemical staining reactions, paraffin sections from a canine oral squamous cell carcinoma were used.

### RNA Isolation and cDNA Synthesis

Total RNA of the EGFP and EGFP-HMGA2 expressing as well as native CT1258 cells were isolated using the NucleoSpin miRNA (Macherey-Nagel, Düren, Germany) kit according to the manufacturer’s instructions including an on column DNase digest to remove potential genomic DNA contaminations.

The respective cDNA syntheses with mRNAs as template were performed using 250 ng total RNA of each sample and the QuantiTect Reverse Transcription Kit following the manufacturer’s protocol (Qiagen, Hilden, Germany).

For the reverse transcription of the miRNAs 30 ng total RNA of each sample, the TaqMan MicroRNA Reverse Transcription Kit and the reverse transcription primer provided with the TaqMan MicroRNA Assays were used. All steps were carried out following the manufacturer’s protocol (Applied Biosystems, Darmstadt, Germany).

### *HMGA1, HMGA2, SNAI1, SNAI2* and *CDH1* Real-time PCR

For relative quantification of the *HMGA2, HMGA1, SNAI1, SNAI2* and *CDH1* transcript levels in relation to the endogenous gene controls *GUSB* and *HPRT1* PCR amplifications were carried out using the Eppendorf Mastercycler ep realplex real-time PCR System (Eppendorf AG, Hamburg, Germany).

2 µl of each cDNA corresponding to 25 ng of total RNA were amplified in a total volume of 20 µl using the TaqMan Universal PCR Master Mix (Applied Biosystems, Darmstadt, Germany) with 600 nM of each primer and 200 nM fluorogenic probe for canine *HMGA1, HMGA2* gene expression analysis (previously published by us in Joetzke et al. [18]. Commercially available TaqMan gene expression assays were used for the analysis of the canine targets *SNAI1* (Cf02705362_s1), *SNAI2* (Cf02701218_u1) and *CDH1* (Cf02697525_m1) as well as for the endogenous controls, canine *GUSB* (Cf02622808_m1) and canine *HPRT1* (Cf02626258_m1) (Applied Biosystems, Darmstadt, Germany).

PCR conditions were as follows: 2 min at 50°C and 10 min at 95°C, followed by 40 cycles with 15 s at 95°C and 1 min at 60°C.

All samples were measured in triplicate and for each run non-template controls and non-reverse transcriptase control reactions were included. A precedent efficiency analysis of all PCR assays used in this study was performed by applying the same template in different dilution steps covering a magnitude of five (cDNA corresponding to 100–0.001 ng RNA). The PCR reactions of all analysed target genes showed comparable efficiencies ensuring an appropriate relative real-time PCR analysis. For the analysis based on ΔΔCT method native CT1258 cells were defined as calibrator.

### *Let-7a*, *RNU6B* Real-time PCR

Relative quantification of the canine *let-7a* and *RNU6B* miRNA transcript levels were carried out using the Eppendorf Mastercycler ep realplex real-time PCR System (Eppendorf AG, Hamburg, Germany). 1.33 µl of each cDNA were amplified in a total volume of 20 µl using TaqMan Universal PCR Master Mix (Applied Biosystems, Darmstadt, Germany), No AmpErase UNG and TaqMan MicroRNA assays for *let-7a* (Assay ID: 000377) and *RNU6B* (Assay ID: 001093) (Applied Biosystems, Darmstadt, Germany).

PCR conditions were as follows: 10 min at 95°C, followed by 40 cycles with 15 s at 95°C and 1 min at 60°C.

All samples were measured in quadruplicate and for each run non-template controls and non-reverse transcriptase control reactions were included.

A precedent efficiency analysis of the miRNA PCR assays which were used in this study was performed by applying the same template in different dilution steps, showing comparable efficiencies. For the analysis based on ΔΔCT method the control group was defined as calibrator performing relative real-time PCR with *let-7a* as target gene.

### Real-time PCR Statistical Analysis

Statistical analysis of the relative real-time PCR results was performed applying the hypothesis test with the software tool REST 2009, version 2.0.13 (Qiagen, Hilden, Germany) [50]. REST determines whether there is a significant difference between samples and controls taking into account reaction efficiencies and using randomisation techniques. A p-value of ≤0.05 was considered to be statistically significant.

### Cell Proliferation Assay

The proliferation of native CT1258 cells in comparison to the established fluorescent CT1258-EGFP and CT1258-EGFP-HMGA2 cell lines was evaluated using a colorimetric BrdU cell proliferation ELISA (Roche Applied Science, Mannheim, Germany). This assay measures the incorporation of the thymidine analogue 5-bromo-2-deoxyuridine (BrdU) into newly synthesised DNA of replicating cells by ELISA using an anti-BrdU monoclonal antibody.

A total number of 15.000 cells/well from each CT1258 cell line was seeded in eight different wells and cultivated at the previously described conditions. BrdU was added after 24 h and incubated for two hours. The proliferation assay was carried out according to manufacturer’s protocol (Cell proliferation ELISA, colorimetric, Roche Applied Science, Mannheim, Germany). The reaction products were quantified by measuring the absorbance at 370 nm (reference wavelength 492 nm) with a maximum of 27 single reads over a time period of 30 min using a scanning multi-well spectrophotometer equipped with the analysis software Gen 5 (Synergy HT multi-mode microplate reader, BioTek Instruments Inc., Bad Friedrichshall Germany). The absorbance results directly correlate to the amount of DNA synthesis and hereby to the number of proliferating cells.

Results are stated as mean absorbance values expressed as Max V [delta 370–492] and presented as mean ± standard deviation. All statistical analyses were performed using OriginPro 8 software (OriginLab Corporation, Northampton, USA). The Shapiro-Wilk test was applied to test if the data are normally distributed. Based on the outcome of the Shapiro-Wilk test, a paired sample t-test was performed to assess the significance of proliferative differences between CT1258-EGFP, CT1258-EGFP-HMGA2 and native CT1258 cells. Differences were considered statistically significant for * p≤0.05, ** p≤0.001 to 0.01 and *** p<0.001.

### Chromosome Preparation

For chromosome preparation of CT1258-EGFP and CT1258-EGFP-HMGA2 cells colcemid (Biochrom AG, Berlin, Germany) was added at a final concentration of 0.1 µg/ml for 90 min before harvesting. Subsequently, the cells were incubated for 20 min in hypotonic medium (1: 6; medium 199: H_2_O; (medium 199: Life Technologies GmbH, Darmstadt, Germany)) and finally fixed with methanol/glacial acetic acid (3∶1) following routine methods [51]. The suspension was dropped on ice-cold slides and dried for 5 days at 37°C followed by GTG-banding which was performed as previously described by [52]. Results were processed and recorded with BandView, 6.0, MultiSpecies, Applied Spectral Imaging, Israel. Karyotype description followed the nomenclature proposed by Reimann et al. [53].

## Results

### Fluorescence Microscopy and FCM

#### Fluorescence microscopy

**Figure 1:**
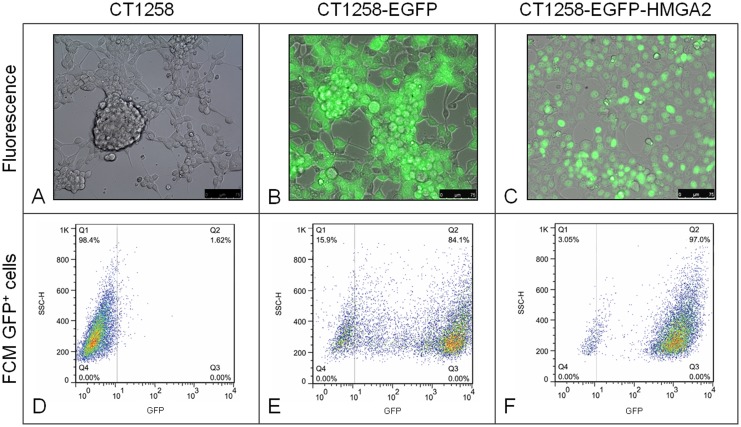
Fluorescence microscopy and flow cytometry analyses. A: Transmitted light image of the characteristic growth pattern of native CT1258 cells. B: Merged image of EGFP expressing CT1258-EGFP cells; EGFP is localised in the cytoplasm. C: Merged image of CT1258-EGFP-HMGA2 cells expressing the nuclear localised EGFP-HMGA2 fusion protein. D–F: Flow cytometric analyses of EGFP expression in the three CT1258 cell lines depicted in dot-plots showing side scatter (SSC) vs. EGFP fluorescence. No GFP fluorescence is detectable in native CT1258 cells (D), 84.1% of EGFP-positive cells are present in the CT1258-EGFP cell line and (F) 97.0% EGFP-positive cells in CT1258-EGFP-HMGA2. Per sample 1×10^4^ events were analysed.

CT258 cells transfected with the non-recombinant pEGFP-C1 expression vector showed green fluorescence all over the cytoplasm due to EGFP expression (Fig. 1B) whereas unmodified native CT1258 cells showed no EGFP fluorescence (Fig. 1A).

Transfection of CT1258 with pEGFPC1-HMGA2 resulted in the expression of a recombinant canine EGFP-HMGA2 fusion protein which could solely be detected in the nucleus of the transfected cells (Fig. 1C).

#### FCM

For determination of EGFP positive cells by FCM, both fluorescent cell lines were compared to native non-transfected CT1258 cells (Fig. 1D). Dead, TO-PRO-3 positive cells were eliminated by gating prior to the EGFP positivity analysis. The cells were measured for CT1258 in the 319th passage, for CT1258-EGFP in the 27th passage, and for CT1258-EGFP-HMGA2 in the 113th passage.

The vitality of the cell lines ranged from 85% to 93% (data not shown). A mean percentage of 84.1% EGFP positive cells from the total cell population of the G418 selected CT1258-EGFP cell line (Fig. 1E) and 97.0% EGFP positive cells for the CT1258-EGFP-HMGA2 cell line (Fig. 1F) was determined.

### Immunocytochemistry

**Figure 2:**
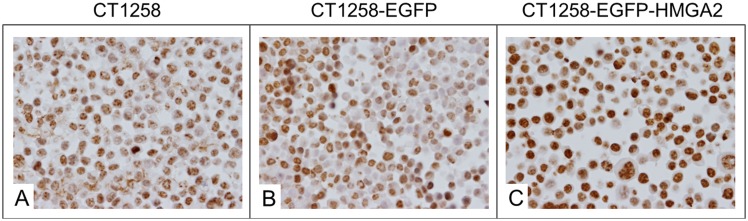
Immunocytochemical staining. A: Native CT1258 cells, B: CT1258-EGFP cells, C: CT1258-EGFP-HMGA2 cells. Approximately 50% of the native CT1258 cell line and of CT1258-EGFP cells showed a HMGA2-positive nuclear labelling. In approximately 70–80% of CT1258-EGFP-HMGA2 cells, a strong and exclusively nuclear labelling for HMGA2 was detectable.

Approximately 50% of the CT1258-EGFP cell line had nuclear labelling for HMGA2 (Fig. 2B). In approximately 70–80% of CT1258-EGFP-HMGA2 cells strong labelling for HMGA2 was detected, which was exclusively present in the nucleus (Fig. 2C).

### Relative *HMGA2* Real-time PCR Expression Analysis

**Figure 3:**
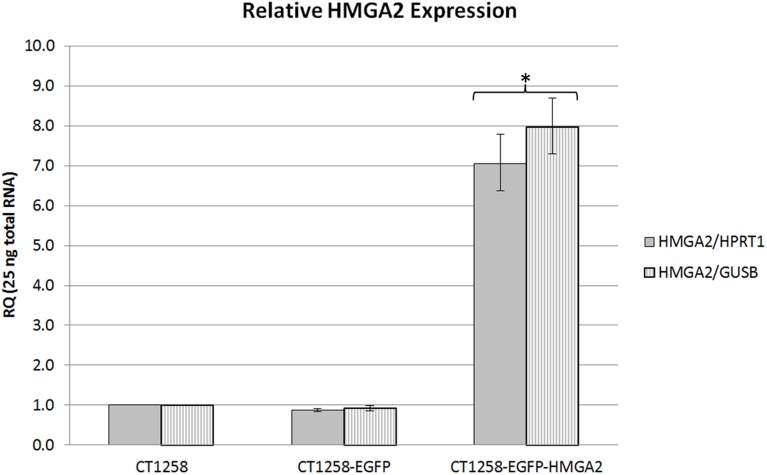
*HMGA2* real-time PCR analyses. Relative *HMGA2/HPRT1* and *HMGA2/GUSB* expression in native CT1258, CT1258-EGFP and CT1258-HMGA2-EGFP cells. Error bars are standard deviations. *p≤0.05 indicates a statistical significant expression deregulation of *HMGA2* in CT1258-HMGA2-EGFP cells when compared to native CT1258.

All real-time PCR results were analysed based on ΔΔCT method. The expression ratio of *HMGA2* mRNA in CT1258-EGFP cells was found to be 0.88/0.92 relative to *HPRT1/GUSB* expression when compared to the level seen in native CT1258 cells (Fig. 3). In contrast, the *HMGA2* expression in CT1258-EGFP-HMGA2 cells was 7.0/8.0 fold increased (relative to *HPRT1/GUSB*) when compared to the respective expression in native CT1258 cells (Fig. 3).

### Relative *Let-7a* Real-time PCR Expression Analysis

**Figure 4:**
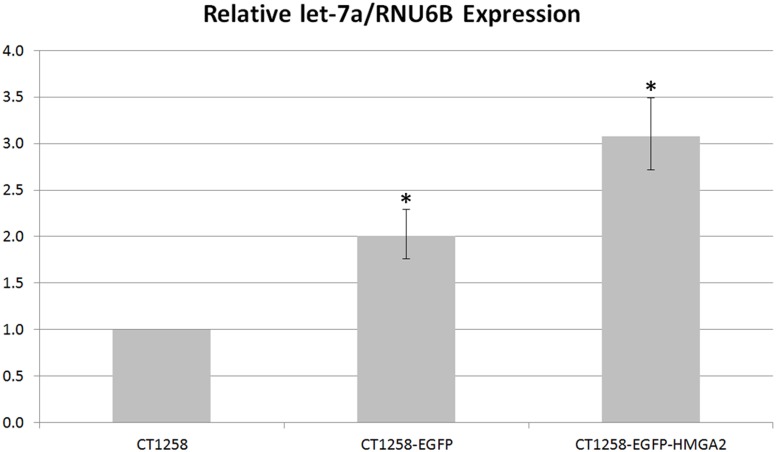
*Let-7a* real-time PCR analyses. Relative *let-7a*/RNU6B expression in native CT1258, CT1258-EGFP and CT1258-HMGA2-EGFP cells. Error bars are standard deviations. No statistical significant expression deregulation of *let-7a* in CT1258-EGFP and CT1258-HMGA2-EGFP was detected when compared to native CT1258 cells. Statistical significant p value was defined as ≤0.05.

The *let-7a* expression level in CT1258-EGFP and CT1258-EGFP-HMGA2 cells was 2.0 and 3.1 fold higher (relative to *RNU6B*) when compared to the detected expression in native CT1258 cells (Fig. 4).

### Relative *HMGA1* Real-time PCR Expression Analysis

**Figure 5:**
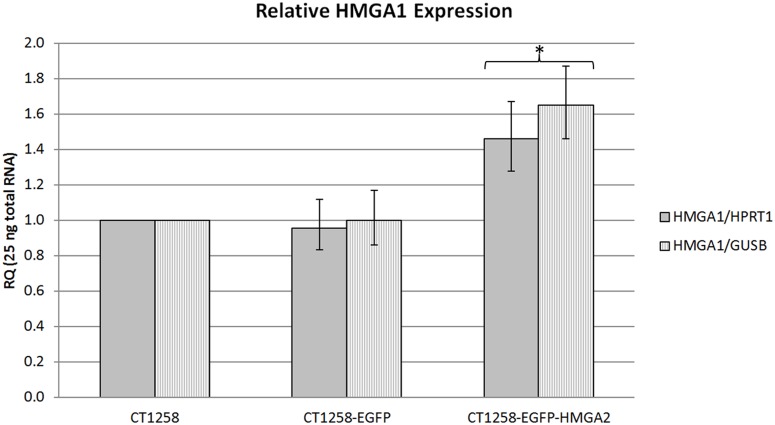
*HMGA1* real-time PCR analyses. Relative *HMGA1/HPRT1* and *HMGA1/GUSB* expression in native CT1258, CT1258-EGFP and CT1258-HMGA2-EGFP cells. Error bars are standard deviations. *p≤0.05 indicates a statistical significant increased expression of *HMGA1* in CT1258-HMGA2-EGFP cells when compared to native CT1258 and CT1258-EGFP.

The *HMGA1* level was 1.5 and 1.7 fold increased (relative to *HPRT1* and *GUSB*) in CT1258-EGFP-HMGA2. In CT1258-EGFP cells a comparable increased expression could not be detected (1.0/1.0 relative to *HPRT1* and *GUSB*) when compared to the native cells (Fig. 5).

### Relative *SNAI1, SNAI2* and *CDH1* Real-time PCR Expression Analysis

Relative *SNAI1* expression to the housekeeping genes *HPRT1/GUSB* was found to be 0.8/0.8 respectively in CT1258-EGFP and 1/1.2 in the CT1258-EGFP-HMGA2 when compared to native cells CT1258 (figure S1).

Relative *SNAI2* expression (relative to *HPRT1/GUSB*) was found 1.5/1.6 in CT1258-EGFP cells and 1.4/1.6 in CT1258-EGFP-HMGA2 cells when compared to CT1258 (figure S2).

*CDH1* was barely expressed in all cell lines with Ct values higher than 36, thus an analysis by the ΔΔCT method was not possible.

### Real-time PCR Statistical Analysis

The hypothesis test of the relative real-time PCR results was performed using REST software tool 2009, version 2.0.13 (Qiagen, Hilden, Germany) [50]. The statistical analyses were carried out separately for the CT1258-EGFP and CT1258-EGFP-HMGA2 cells in comparison to native CT1258. A p-value of≤0.05 was considered as statistically significant.

The statistical analysis showed no significant differences of the relative *HMGA2* expression in the CT1258-EGFP cells in comparison to native CT1258 cells (p = 0.075) (Fig. 3). The CT1258-EGFP-HMGA2 cell line showed a significant *HMGA2* over-expression in comparison to native CT1258 cells (p = 0.009) and CT1258-EGFP cells (p = 0.000) (Fig. 3).

The relative *let-7a* expression differed significantly in CT1258-EGFP (p = 0.003) and CT1258-EGFP-HMGA2 (p = 0.012) compared to the native CT1258 cells (Fig. 4). The additional statistical analysis of the *let-7a* expression between the CT1258-EGFP and CT1258-EGFP-HMGA2 cells showed also statistical significance (p = 0.021).

The *HMGA1* showed no statistical difference in CT1258-EGFP (p = 0.087) but a significantly higher expression level in CT1258-EGFP-HMGA2 in comparison to the native cell line CT1258 (p = 0.000) and the CT1258-EGFP cells (p = 0.000) (Fig. 5).

*SNAI1* expression was statistically significantly different in CT1258-EGFP in comparison to the *SNAI1* levels in the native cell line (p = 0.016). In CT1258-EGFP-HMGA2 the *SNAI1* expression was comparable to native CT1258 cells (p = 0.462) (figure S1).

*SNAI2* expression of CT1258-EGFP (p = 0.100) and CT1258-EGFP-HMGA2 (p = 0.066) were both not significantly different in comparison to the native cell line (figure S2). For *CDH1* expression no statistical analyses was performed due to barely detectible or absent gene expression.

### Cell Proliferation Assay

The proliferation of the two established fluorescent CT1258 cell lines and native CT1258 cells was measured using a BrdU proliferation test to analyse the effect of EGFP-HMGA2 expressed in CT1258-EGFP-HMGA2 cells.

**Figure 6:**
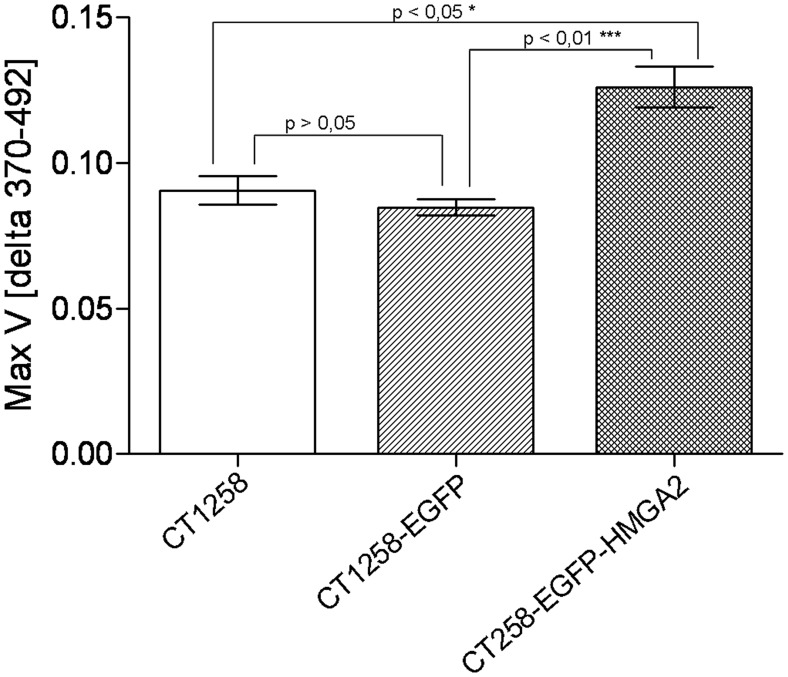
BrdU cell proliferation assay. Measured proliferation of native CT1258, CT1258-EGFP and CT1258-HMGA2-EGFP cells. A statistical significant increased proliferation was detected for CT1258 cells expressing the EGFP-HMGA2 fusion protein in comparison to native CT1258 and EGFP expressing CT1258 cells. Each bar represents a mean ± SD, *p≤0.05, ***p≤0.001.

The proliferation of each cell line was compared with the two other cell lines (Fig. 6). A significantly increased cell proliferation activity with a p-value of <0.05 was ascertained for CT1258-EGFP-HMGA2 cells in comparison to native CT1258 cells. Comparing CT1258-EGFP-HMGA2 cells vs. CT1258-EGFP cells resulted also in significantly increased cell proliferation for CT1258 expressing EGFP-HMGA2, but with a p-value of <0.01. The analysis of native CT1258 vs. CT1258-EGFP cells resulted in no significant proliferative differences (p>0.05) between both cell lines.

### Cytogenetic Analyses

**Figure 7:**
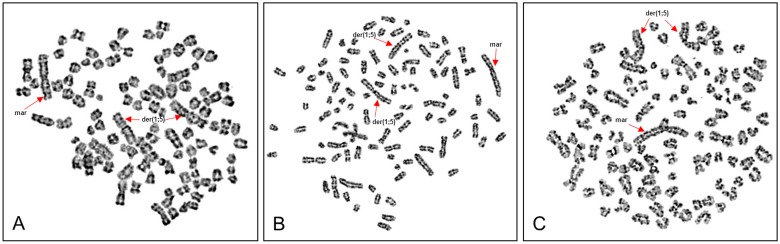
Cytogenetic analyses. Metaphase spreads derived from CT1258 (A), CT1258-EGFP (B) and CT1258-EGFP-HMGA2 (C) cells after GTG-banding. The arrows indicate the centromeric fusions between the canine chromosomes 1 and 5 (der(1;5)) and a large bi-armed marker (mar) consisting of material from chromosomes 1 and 2 being characteristic for the CT1258 cell line.

**Figure 8:**
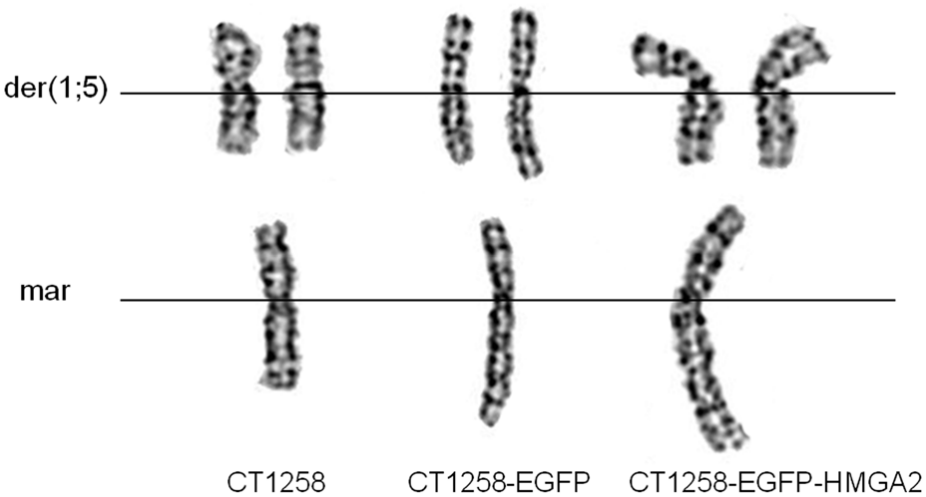
Details of CT1258 chromosomal aberrations. Detailed presentation of chromosomal aberrations found in metaphases of CT1258, CT1258-EGFP and CT1258-EGFP-HMGA2. Two derivative chromosomes (der(1;5)) and the large bi-armed marker chromosome (mar) were found in each cell line.

The analysis of native CT1258 cells revealed the presence of a hyperdiploid karyotype (Fig. 7A). Centromeric fusions between the canine chromosomes 1 (CFA1) and 5 (CFA 5), in the following named as der(1;5), were present (Fig. 8). Additionally, one large bi-armed marker (mar) consisting of material from chromosomes 1 and 2 was found (Fig. 8). The gained results are comparable to our previous cytogenetic analysis of primary CT1258 cells carried out by Winkler *et al.* in 2005 [45] concerning the present der(1;5) and the bi-armed marker chromosome (mar). In contrast, native CT1258 cells showed no longer the centric fusions of chromosomes 4 (CFA4) and 5 (CFA5) (named der(4;5)) as described in 50% of the initially analysed metaphases of primary CT1258 cells [45].

The chromosome analyses of CT1258-EGFP and CT1258-EGFP-HMGA2 revealed a comparable hyperdiploid karyotype as described for native CT1258 cells (Fig. 7B, 7C). The same large bi-armed marker chromosome (mar) and the two der(1;5) chromosomes as described for native CT1258 cells were also present (Fig. 7B, 7C and Fig. 8).

## Discussion

Re-expression of *HMGA2* is reported to be associated with the formation of malignant and benign tumours [17]–[25], [54]–[57], but the exact mechanism of HMGA2 acting in tumour formation and progression is still unclear. In general, miRNA *let-7* family members are regulating *HMGA2* time-dependently in a negative way by binding to multiple target sites in the 3′UTR of *HMGA2* mRNA [31]–[34], [39]. In different tumours, the *HMGA2* 3′UTR was described to be affected by deletions or rearrangements [58] leading to a loss of *let-7* complementary target sequences [39], [59]. A truncated *HMGA2* mRNA without *let-7* binding sites escapes the *let-7* regulation resulting in increased expression of HMGA2 protein [31], [32], [39]. Generally, a delicate balance of *let-7* and *HMGA2* is discussed to be necessary for cells to switch between undifferentiated and differentiated state and also plays a central role in cancer development and progression [33], [60]–[63]. How HMGA2 exerts these changes is not completely understood.

Within this study, we analysed the described *let-7*-*HMGA2* regulation mechanism in canine prostate cancer using the naturally HMGA2-overexpressing canine adenocarcinoma derived cell line CT1258 as an *in vitro* model.

The CT1258 cell line was as previously described to be derived from an aggressive canine prostate carcinoma [45]. In previous studies we characterised the *in vivo* behaviour and tumour formation capacity of CT1258 in NOD/SCID [47], [48]. Herein, it could be shown that a very low number of 1×10^3^ subcutaneously injected CT1258 cells [47] and an intraperitoneal inoculation of 1×10^5^ cells was sufficient to induce stable tumour growth [46]. The induced tumours showed highly aggressive growth mimicking the character of the original neoplasia [46], [47]. Comparative analyses of the primary neoplasia, the initial established CT1258 cell line and the CT1258 generated tumours showed that the cell line and the induced tumours kept their characteristics including cytogenetics, marker expression and in case of the induced tumours the histopathological presentation [45]–[47].

Thus, the native CT1258 cell line provides a well-characterised basis to identify and characterise molecular mechanisms playing a key role in prostate cancer.

With the recombinant CT1258-EGFP-HMGA2 cell line, expressing an EGFP-HMGA2 transcript lacking the 3′UTR, we investigated if the proliferative effect of HMGA2 can even be further enhanced although the endogenous *HMGA2* mRNA level in native CT1258 cells is already highly elevated compared to non-neoplastic prostate tissue (unpublished data). Moreover, we analysed the potential impact of the ectopic HMGA2 expression on the miRNA *let-7a* as one of its regulators within the CT1258-EGFP-HMGA2 cell line in comparison to native CT1258 cells and the control cell line CT1258-EGFP.

In addition the gene expression of the direct HMGA2 target genes *HMGA1*, *SNAI1, SNAI2* and the downstream target *CDH1* were examined [48], [49].

Verification using fluorescence microscopy detected high numbers of EGFP-positive cells expressing either the cytoplasmatic EGFP protein localised throughout the cell or the EGFP-HMGA2 fusion protein. The EGFP-HMGA2 protein was shown to be accumulating exclusively into the nucleus as known for the native protein.

The nuclear accumulation of the recombinant EGFP-HMGA2 fusion protein represents HMGA2-typical characteristics such as a functional nuclear localisation signal and chromatin-binding properties enabling proper EGFP-HMGA2 protein function. Further, an irregular distribution of EGFP-HMGA2 amongst the chromatin could be observed matching previous reports characterising native HMGA2 by other groups [49], [60], [64], [65]. This irregular nuclear distribution of HMGA2 could also be shown by our immunocytochemistry analyses. The results of the HMGA2 immunocytochemistry revealed a distinct nuclear labelling in approx. 50% of native and CT1258-EGFP cells, while 70–80% of the CT1258-EGFP-HMGA2 cells showed a strong nuclear labelling. Due to the strong nuclear signal in the CT1258-HMGA2-EGFP cells showing the same irregular labelling as seen by fluorescence microscopy, the presence and the functionality of the ectopically expressed HMGA2-GFP fusion protein could be detected via both methodologies.

The flow cytometry analyses confirmed the observed high numbers of fluorescent cells resulting in 84.1% CT1258-EGFP and 97.1% CT1258-EGFP-HMGA2 positive cells expressing EGFP. Thus, the antibiotic selection with G418 showed as very effective to generate nearly pure recombinant derivatives of the CT1258 cell line which can be used as a tool for subsequent *in vivo* experiments.

Real-time PCR analyses of *HMGA2* expression revealed comparable *HMGA2* levels in native CT1258 and CT1258-EGFP cells while a statistically significant *HMGA2* overexpression could be detected in the CT1258-EGFP-HMGA2 cell line. This leads to the assumption that by transfection of CT1258 cells with the pEGFP-C1-*HMGA2* expression vector construct and selection with G418 an ectopic EGFP-HMGA2 expression could be successfully implemented.

Furthermore, *let-7a* real-time PCR expression analyses were performed to investigate potential connections on the *HMGA2-let-7*-axis in canine prostate cancer. The results showed significantly increased *let-7a* expression levels in CT1258-EGFP and CT1258-EGFP-HMGA2 in comparison to the native cell line, whereupon the highest *let-7a* level was detected in CT1258-EGFP-HMGA2. The CT1258-EGFP cell line was intended to serve as a control cell line to exclude EGFP-induced effects. Thus, a comparable *let-7a* expression was expected in native CT1258 cells and the CT1258-EGFP cell line. Interestingly, significantly higher *let-7a* levels were found in CT1258-EGFP. This might be explained by off-target effects induced by the treatment with G418 or the unidentified integration loci of the expression vector into the genome potentially affecting *let-7a* regulatory sites. An effect of EGFP overexpression on the *let-7a* expression is unlikely as EGFP was used as a reporter protein within previously published *let-7* studies showing no EGFP-induced side-effects on *let-7* expression [66], [67].

As described previously, the recombinant inserted canine *HMGA2* CDS in the CT1258-HMGA2-EGFP cell line is lacking the 3′UTR which was expected to result in an escape of the recombinant transcript from the *let-7a* miRNA suppression [31]. Owning to the fact that several protein products encoded by the *let-7a-*regulated mRNAs as e.g. *Lin-28*, *Dicer*, *Myc* and *Argonaute* [68]–[71] were reported to constitute a feedback loop with its regulator, we hypothesized if a HMGA2 protein overexpression might influence the *let-7a* level as well. However, a significantly higher expression of *let-7a* was detected not only in CT1258-EGFP-HMGA2 but as well in the CT1258-EGFP control cell line. Further statistical analysis revealed that the expression of *let-7a* in CT1258-EGFP-HMGA2 was not only significantly higher in comparison to native cells but also in comparison to the CT1258-EGFP control cell line. It seems that the cells responded to the elevated levels of the recombinant HMGA2 with increased *let-7a* expression. However, the elevated *let-7a* levels can not solely be attributed to a direct feedback loop as the one previously described for the above mentioned *let-7* targets [68], [70], [71]. An alternative, indirect response of the cells which are “sensing” the HMGA2 overproduction or unspecific plasmid DNA integration into the genome might be possible as well. Although the stimulated *let-7a* expression by the ectopic HMGA2 overexpression was not entirely proofed within this study the newly generated CT1258-EGFP-HMGA2 cell line is nevertheless a suitable tool to further investigate the impact of HMGA2 expression on other *HMGA2* regulating and regulated genes in canine prostate cancer.

To further examine the role of ectopically overexpressed HMGA2, the expression of the HMGA2-regulated targets *HMGA1*, *SNAI1*, *SNAI2* and the downstream target *CDH1* was analysed. These targets are of considerable interest as they were described to be involved in the EMT and thus are able to promote the invasion, migration and subsequent metastasis of prostate cancer cells [72], [73]. The analyses of the *HMGA2*-related family member *HMGA1* revealed a potential positive regulation by the overexpression of the HMGA2-EGFP fusion protein. We could show that *HMGA1* was significantly higher expressed in CT1258-EGFP-HMGA2 cells compared to native CT1258 and the CT1258-EGFP control cell line. Interestingly, the *HMGA1* transcript is also described to be negatively regulated by the same *let-7* mechanism as previously described for *HMGA2* [35], [74]. In accordance with our results, Berlingieri *et al.* described in a previous study a positive, HMGA2-dependent regulation of *HMGA1* in rat thyroid cells [48].

The analysis of the other HMGA2 targets *SNAI1, SNAI2* and its negatively regulated downstream target *CDH1* [49] showed no differences in expression except *SNAI1*. *SNAI1* was significantly lower expressed in the CT1258-EGFP but not significantly different in CT1258-EGFP-HMGA2 compared to native CT1258 cells.

The cell proliferation analyses by BrdU incorporation assay showed that the ectopic overexpression of recombinant HMGA2-EGFP in the CT1258-HMGA2-EGFP cell line resulted in a significantly increased cell proliferation in comparison to native CT1258 and CT1258-EGFP cells. The results revealed that native CT1258 and CT1258-EGFP cells presented nearly the same proliferative rate and thereby excluding that a cell proliferative effect might be mediated by EGFP expression. Consequently the seen effect can be attributed to the ectopic overexpression of HMGA2 within the CT1258-HMGA2-EGFP cell line.

The present results are in accordance with other studies where ectopic overexpression of recombinant HMGA2 was also shown to have a positive effect on cell proliferation in e.g. rat fibroblasts [75], or murine myeloblasts [76] *in vitro* and on hematopoietic tissue derived from transgenic HMGA2-overexpressing mice [59]. The comparability of these pervious results and the proliferative characteristics of CT1258-HMGA2-EGFP underline the functionality of the introduced recombinant protein.

The cytogenetic analyses of the recombinant fluorescent cell lines CT1258-EGFP and CT1258-HMGA2-EGFP revealed stable chromosome copy numbers resembling the hyperdiploid karyotype with der(1;5) chromosomal fusions and the characteristic large bi-armed marker chromosome mainly consisting of material from CFA1 and CFA2 found in native CT1258 cells. The karyotype of CT1258 native cells and their fluorescent derivatives has changed slightly compared to cells of CT1258, which were analysed in a very early passage by Winkler et al. in 2005 [45]. In addition to the marker chromosome and the der(1;5) chromosome, the centric fusion of CFA4 and CFA5 (der(4;5)) found in 50% of the analysed metaphases of primary CT1258 cells [45] was no longer present in the native CT1258 cells used in the present study. This loss of der(4;5) can probably be explained due to selection in direction to der(1;5) during the cultivation of the cells over time as the der(4;5) was only found in 50% of the primary analysed CT1258 cells. With the comparative cytogenetic we could assure that no macroscopic chromosomal aberrations such as fusions or breakpoints were induced by the transfection and subsequent integration of the expression vectors pEGFP-C1 and pEGFP-C1-*HMGA2* into the genome under G418 antibiotic selection pressure.

Cell lines represent a key tool in cancer research allowing investigating complex interrelations of certain target genes in tumour development in vitro in basic research experiments. With the newly established canine CT1258-EGFP-HMGA2 cell line we could demonstrate in vitro an increased cell-proliferative effect of ectopic overexpressed EGFP-HMGA2. Moreover, the generated data adds functional data helping to understand the complex regulation mechanisms between *HMGA2*, *let-7a* and further selected targets in the progression of prostate cancer.

This CT1258-EGFP-HMGA2 cell line provides a valuable tool to further decipher the HMGA2-mediated molecular mechanisms of prostate cancer and to identify potential targets for development of novel therapies.

Additionally, the ability of the CT1258-EGFP-HMGA2 cell line to express an enhanced EGFP tagged HMGA2 fusion protein can be utilised to monitor the *in vivo* behaviour of the cell line using fluorescence imaging subcutaneously.

To further extend the presented in vitro findings, *in vivo* studies need to be carried out. In perspective, this could allow to characterise if abundantly expressed recombinant HMGA2 can increase the highly tumourigenic potential of CT1258 which was previously demonstrated in a murine NOD/SCID *in vivo* model [46], [47]. The first characterisation of this hypothesis needs to be carried out carefully in an intermediary *in vivo* mouse model. Such an HMGA2-overexpressing *in vivo* mouse model will help to elucidate, if the previously described correlation between HMGA2 and the malignant and metastatic potential of prostate cancer [17] can be reflected and to characterise the underlying molecular mechanisms. Based on this, novel therapeutic options can be established within an *in vivo* mouse model and subsequently applied to treat dogs being affected by prostate cancer.

Xenograft mouse models with implanted human prostate cancer cell lines such as LNCaP [77], PC-3 [78] or DU145 [79] are extremely useful to study the biology of prostate cancer and are used routinely in human research to evaluate prostate cancer therapies. However, xenografts mouse models miss some important characteristics of naturally occurring tumours which experimentally induced tumours or tumours transplanted into immunocompromised animals cannot provide and bear limitations concerning metabolism, body size and age [80], [81]. Thus, long term disease studies are difficult to accomplish within mouse models due to a short life span in comparison to humans [82]. Since prostate cancer develops in dogs spontaneously under the surveillance of an intact immune system in a syngeneic host and tumour microenvironment [83], the dog as a companion animal model provides an important translational bridge between the mouse xenografts and human clinical trials [10], [84]. In fact, dogs were suggested by the National Cancer Institute as a potential population to incorporate into studies of new therapeutics [84], [85].

Consequently, the dog’s contribution to translational research provides reciprocal benefit for both species with the potential to significantly enhance the understanding of prostate cancer development and progression.

## Conclusions

In conclusion, with the herein generated new fluorescent canine CT1258-EGFP-HMGA2 cell line a stable highly reproducible tool for further investigation of HMGA2-mediated cell proliferative effects *in vitro* and *in vivo* in prostate cancer is provided. Screenings as done herein exemplarily for the *HMGA2* regulator *let-7*a and the HMGA2 targets *HMGA1*, *SNAI1*, *SNAI2* and *CDH1* will help to reveal the tumour acting mechanisms. The gained insights of HMGA2-involvement in canine prostate cancer contribute to the identification and evaluation of novel therapeutic options. As the dog displays a unique animal model for prostate cancer, the development of therapeutic strategies provides an important contribution to translational research directed to treat humans, thus providing benefit for both species.

## Supporting Information

Figure S1 ***SNAI1*** **real-time PCR analyses.** Relative *SNAI1/HPRT1* and *SNAI1/GUSB* expression in native CT1258, CT1258-EGFP and CT1258-HMGA2-EGFP cells. Error bars are standard deviations. *p≤0.05 indicates a statistical significant deregulation of *SNAI1* expression in CT1258-EGFP when compared to native CT1258 cells. The CT1258-EGFP-HMGA2 cell line showed no statistical significant different *SNAI1* expression in comparison to native CT1258 cells.
(TIF)

Figure S2 ***SNAI2*** **real-time PCR analyses.** Relative *SNAI2/HPRT1* and *SNAI2/GUSB* expression in native CT1258, CT1258-EGFP and CT1258-HMGA2-EGFP cells. Error bars are standard deviations. No statistical significant deregulation of *SNAI2* expression was detected in CT1258-EGFP and CT1258-HMGA2-EGFP when compared to native CT1258 cells. Statistical significant p value was defined as ≤0.05.
(TIF)
