# Supplementary figures and images for: Generation and Characterisation of a Canine EGFP-HMGA2 Prostate Cancer In Vitro Model
Source: PLoS One. 2014 Jun 10;9(6):e98788. doi: 10.1371/journal.pone.0098788 (PMC4051699; doi:10.1371/journal.pone.0098788)

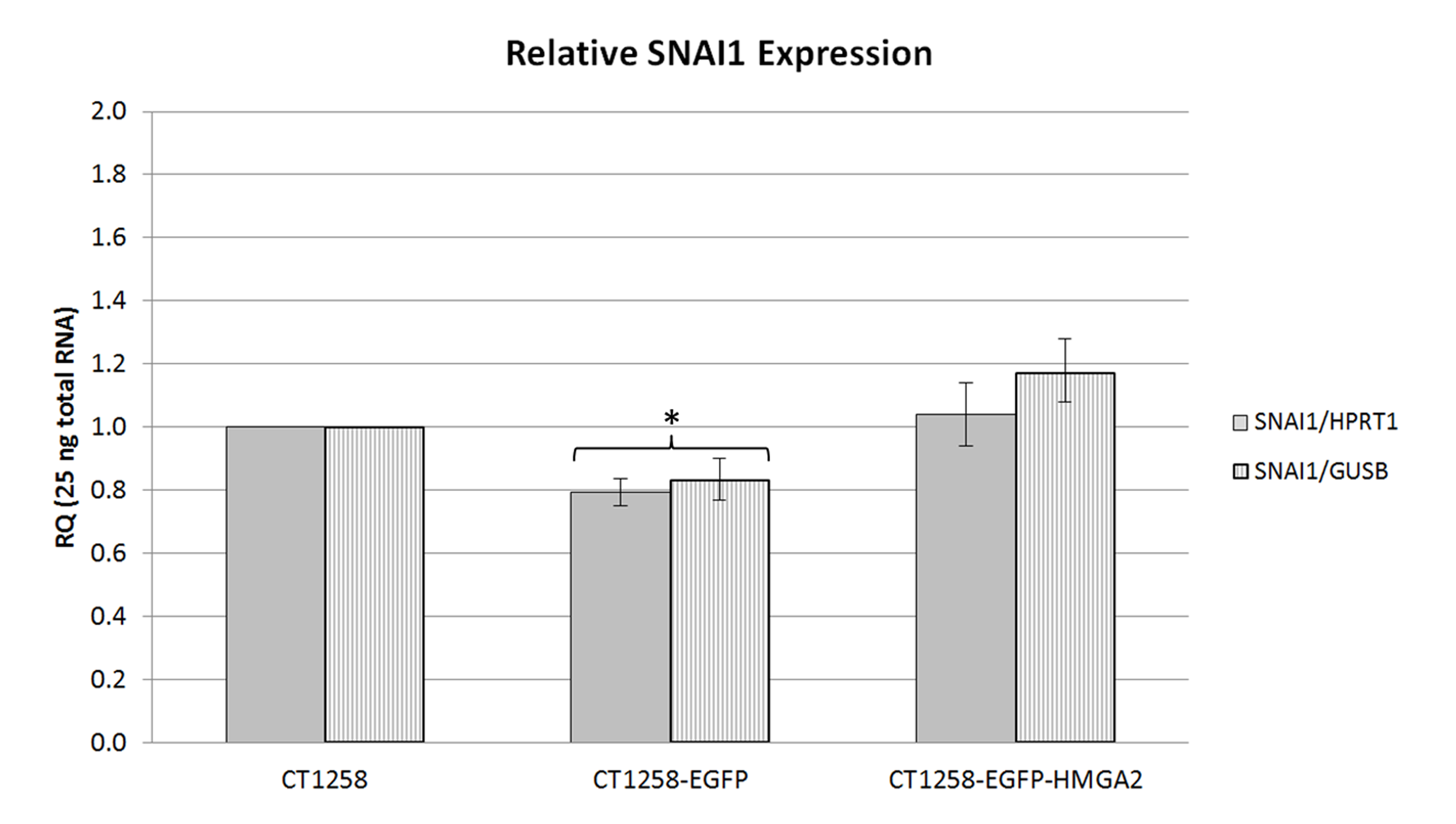

Supplement: Figure S1 — SNAI1 real-time PCR analyses. Relative SNAI1/HPRT1 and SNAI1/GUSB expression in native CT1258, CT1258-EGFP and CT1258-HMGA2-EGFP cells. Error bars are standard deviations. *p≤0.05 indicates a statistical significant deregulation of SNAI1 expression in CT1258-EGFP when compared to native CT1258 cells. The CT1258-EGFP-HMGA2 cell line showed no statistical significant different SNAI1 expression in comparison to native CT1258 cells. (TIF) [file pone.0098788.s001.tif]

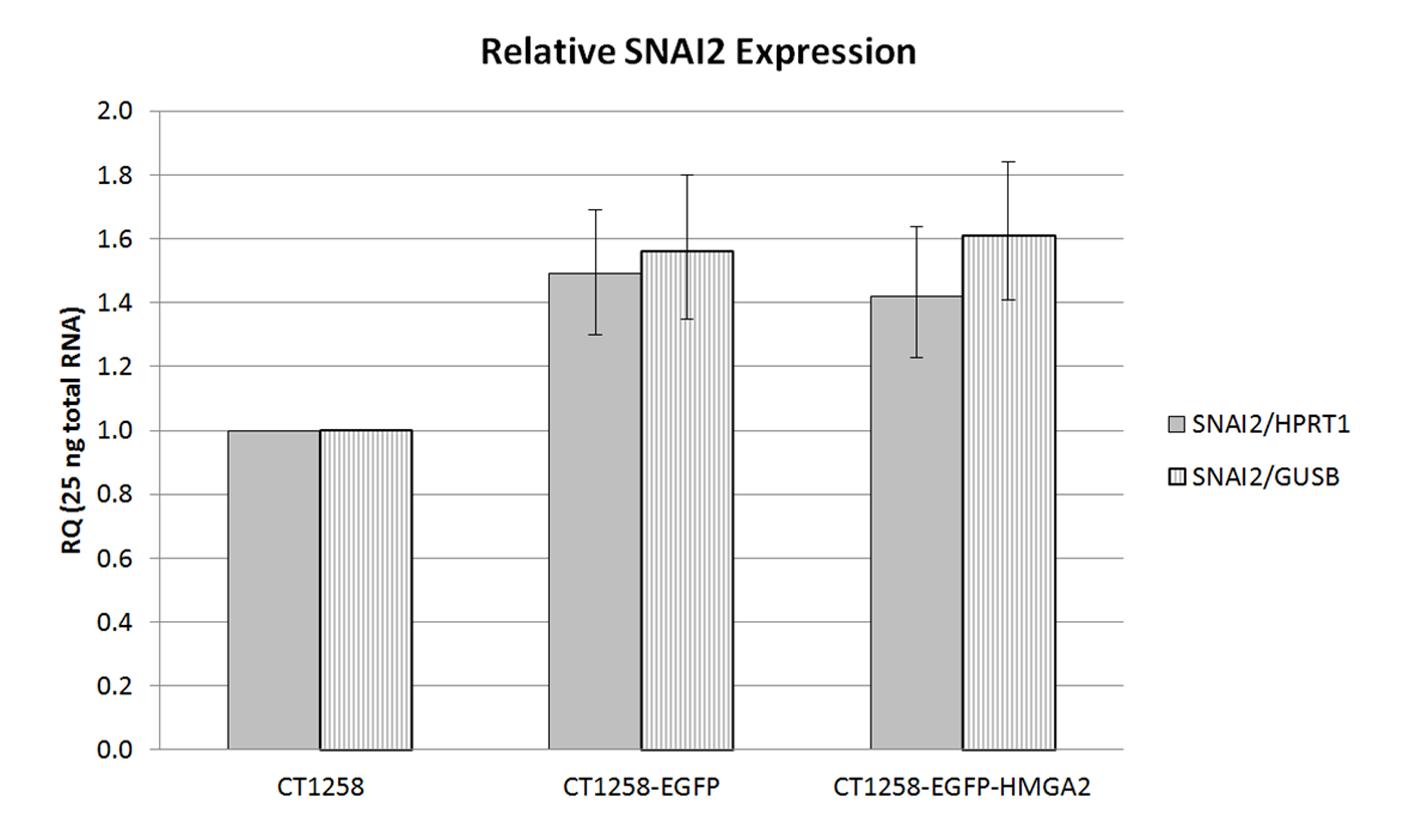

Supplement: Figure S2 — SNAI2 real-time PCR analyses. Relative SNAI2/HPRT1 and SNAI2/GUSB expression in native CT1258, CT1258-EGFP and CT1258-HMGA2-EGFP cells. Error bars are standard deviations. No statistical significant deregulation of SNAI2 expression was detected in CT1258-EGFP and CT1258-HMGA2-EGFP when compared to native CT1258 cells. Statistical significant p value was defined as ≤0.05. (TIF) [file pone.0098788.s002.tif]
